# The effect of fixed and functional remodelling on conduction velocity, wavefront propagation, and rotational activity formation in atrial fibrillation

**DOI:** 10.1093/europace/euae239

**Published:** 2024-09-16

**Authors:** Shohreh Honarbakhsh, Caterina Vidal Horrach, Pier D Lambiase, Caroline Roney, Ross J Hunter

**Affiliations:** William Harvey Research Institute, Queen Mary University of London, Charterhouse Square, London EC1M 6BQ, UK; Electrophysiology Department, Barts Heart Centre, Barts Health NHS Trust, W Smithfield, London EC1A 7BE, UK; William Harvey Research Institute, Queen Mary University of London, Charterhouse Square, London EC1M 6BQ, UK; Electrophysiology Department, Barts Heart Centre, Barts Health NHS Trust, W Smithfield, London EC1A 7BE, UK; William Harvey Research Institute, Queen Mary University of London, Charterhouse Square, London EC1M 6BQ, UK; Electrophysiology Department, Barts Heart Centre, Barts Health NHS Trust, W Smithfield, London EC1A 7BE, UK

**Keywords:** Conduction velocity, Pivot points, Functional remodelling, Scar, Atrial fibrillation

## Abstract

**Aims:**

Pathophysiology of atrial fibrillation (AF) remains unclear. Interactions between scar and conduction velocity (CV) and their impact on wavefront propagation in sinus rhythm (SR) and rotational activity burden in AF were evaluated.

**Methods and results:**

Local activation times (LATs) and voltage data were obtained from patients undergoing ablation for persistent AF. Omnipolar voltage (OV) and bipolar voltage (BV) data were obtained during AF and SR at pacing intervals of 600 and 250 ms. Local activation times were used to determine CV dynamics and their relationship to the underlying voltage and pivot points in SR. Computational modelling studies were performed to evaluate the impact of CVs and fibrosis on rotational activity burden in AF. Data from 60 patients with a total of 2 768 400 LAT and voltage points were analysed (46 140 ± 5689 points/patient). Voltage determined CV dynamics. Enhanced CV heterogeneity sites were predominantly mapped to low-voltage zones (LVZs) (0.2–0.49 mV) (128/168, 76.2%) rather than LVZs (<0.2 mV) and frequently co-located to pivot points (151/168, 89.9%). Atrial fibrillation OV maps correlated better with SR BV 250 ms than 600 ms maps, thereby representing fixed and functional remodelling. Sinus rhythm maps at 250 ms compared with 600 ms harboured a greater number of pivot points. Increased CV slowing and functional remodelling on computational models resulted in a greater rotational activity burden.

**Conclusion:**

Conduction velocity dynamics are impacted by the degree of scar. Conduction velocity heterogeneity and functional remodelling impacts wavefront propagation in SR and rotational activity burden in AF. This study provides insight into the pathophysiology of AF and identifies potential novel ablation targets.

**What’s new**

- In addition to low-voltage zones (LVZs) representing fixed remodelling evident on sinus rhythm (SR) bipolar voltage (BV) 600 ms maps, there were additional LVZs evident on SR BV 250 ms maps representing functional remodelling. Areas of both fixed and functional remodelling demonstrated abnormal CV dynamics together with pivot points during pacing.
- Atrial fibrillation (AF) omnipolar voltage (OV) maps demonstrated additional LVZs not seen on SR BV 600 ms, but a majority were seen on SR BV 250 ms. These data suggest that LVZs on OV maps in AF likely represent a combination of fixed remodelling evident at slower pacing rates, but also functional remodelling only evident at higher pacing rates.
- Computational modelling studies demonstrate that when calibrating to slower CVs and enhanced fibrosis, there is an increase in rotational activity burden in AF. These studies also demonstrated that rotational activity was drawn predominantly to LVZs but also areas of functional remodelling identified only at CLs of 250 ms emphasizing their mechanistic importance in re-entry formation.

## Introduction

Atrial fibrosis, scarring, and electrophysiological remodelling have been proposed as mechanisms that drive and maintain atrial fibrillation (AF). However, the interaction between these in humans has not clearly been evaluated and the impact on wavefront propagation and re-entry formation in AF remains unclear. A greater understanding of this would yield a clearer insight into the pathophysiology of AF and perhaps yield novel targets for substrate modification.

Structural remodelling plays an important role in the pathophysiology of AF, and scar burden has been used as a predictor of procedural outcomes.^1^ Indeed, scar homogenization has been utilized as an ablation strategy for AF with promising results.^2^ However, these studies rely on the premise that all scar is mechanistically important in AF which can lead to extensive ablation often with a modest impact on success rates. Further to this, whether only fixed remodelling is clinically relevant or whether functional remodelling also plays a role in AF remains unclear.

To establish anatomical re-entry activity, slow conduction is required together with areas of scar.^3^ For functional re-entry, heterogeneity in conduction velocity (CV) and/or refractoriness is required. Sites of rate-dependent CV (RDCV) slowing give rise to enhanced CV heterogeneity at shorter cycle lengths (CLs) and have been shown to correlate with micro re-entry activity in atrial tachycardia (AT) and rotational activity in AF.^4,5^ Sites of marked CV heterogeneity have been shown to result in the ability to induce re-entry^3^ and AF.^6^ These studies suggest a potential role for the study of CV behaviour over a range of CLs, often termed CV dynamics to stratify areas of structural remodelling in terms of their mechanistic importance, and RDCV slowing sites as a novel mechanistic marker and potential target for ablation.

The aim of this study was to evaluate the interactions between fixed remodelling, functional remodelling, and CV dynamics in patients with persistent AF and establish how these interactions impact wavefront propagation in sinus rhythm (SR) and formation of rotational activities in AF.

## Methods

Patients undergoing catheter ablation for persistent AF (<24 months and no previous AF ablation) were prospectively included. The exclusion criteria included age <18 years or reversible cause of AF. Patients provided informed consent for their study involvement which was approved by the UK National Research Ethics Service (22/PR/0961). The study was prospectively registered on clinicaltrials.gov (NCT05633303).

### Electrophysiological mapping

EnSite X (Abbott, Chicago, IL, USA) was used as the 3D mapping system. Left atrial (LA) anatomical, voltage, and local activation time (LAT) maps were created using the HD Grid mapping catheter (Abbott).

#### Scar assessment

High-density omnipolar voltage (OV) maps were created in AF (see Supplementary material online, Methods). Omnipolar voltage maps in AF were utilized rather than bipolar voltage (BV) maps in AF, as previous studies have shown that OV maps overcome the underestimation of voltage in AF seen with BV maps as a result of wavefront collision and fractionation in AF and better correlate with SR BV maps.^7,8^ This was to ensure more accurate voltage mapping in AF. Following this, patients underwent direct current cardioversion (DCCV) to SR. Repeat BV maps were created in SR with atrial pacing at pacing intervals (PIs) of 600 and 250 ms. Three voltage zones were defined, very low–voltage zones (vLVZs) < 0.2 mV also referred to as fixed scar, LVZs (0.2–0.49 mV), and non-LVZs (nLVZs), i.e. normal voltage zones ≥0.5 mV.^5^

#### Conduction velocity and conduction velocity dynamic assessment

Local activation time maps created with fixed atrial pacing at PIs of 600, 400, and 250 ms in SR. Local activation time (LAT) maps were created with endocardial proximal and distal coronary sinus pacing and LA appendage pacing to allow different wavefront directionalities to account for the impact of anisotropy and fibre orientation on CV measurements. Each point on the maps obtained for the different pacing sites was assigned a location on the geometry using the individual *xyz* co-ordinates for the point. This thereby ensured the location of each point was accurately transcribed onto the geometry, thereby allowing comparison of the voltage and LAT data between points obtained from the maps created using different pacing sites.

### Ablation approach

Following the LAT maps, patients underwent pulmonary vein isolation (PVI) with bilateral wide area circumferential ablation (WACA) using radiofrequency ablation (see Supplementary material online, Methods).

### Conduction velocity methodology

Conduction velocity was defined as the distance travelled by a wavefront in a unit of time. Conduction velocity was calculated from electroanatomic mapping data consisting of an LA anatomical mesh and LATs at recording locations projected to the atrial surface (see Supplementary material online, Methods).

Conduction velocity measurements were obtained for all three PIs. Differences in the CV measurements at each anatomical location with rate were elicited from these PIs. The voltage at each CV measurement was also determined. To evaluate heterogeneity in CV dynamics, sites of RDCV slowing were identified. These were defined as sites exhibiting a reduction in CV between PI = 600 ms and PI = 250 ms that was ≥20% greater than the mean CV reduction seen between these PIs for other sites of that voltage.

Sites demonstrating differences in CV measurements due to the atrial pacing site were excluded from the analysis to ensure the CV measurements obtained were not impacted by anisotropy.

### Fixed and functional remodelling

The LA body was defined as the LA excluding mitral valve annulus and PVs. Voltage parameters were compared on OV maps in AF (AF OV) and SR BV maps at a PI of 600 ms (SR BV 600 ms) and 250 ms (SR BV 250 ms). Areas of remodelling were defined by voltage as follows: (i) fixed remodelling (voltage <0.5 mV on SR BV 600 ms maps) and (ii) functional remodelling (voltage ≥0.5 mV on SR BV 600 ms maps but <0.5 mV on AF OV and SR BV 250 ms maps).

To review the differences in voltage at an anatomical site on the three voltage maps, voltage points were co-registered in accordance with *xyz* co-ordinates. A point was defined as co-locating to another point if they were within a geodesic distance of <3 mm. The CVs obtained and the anatomical distribution using a six-segment model (anterior, lateral, septal, posterior, inferior, and roof) of sites of voltage discrepancy were evaluated.

### Pivot points

A novel wavefront tracking algorithm developed and executed in MATLAB was used offline to track wavefront propagation in SR (see Supplementary material online, Methods). Unipolar recordings using the HD Grid catheter were collected by referencing to the Wilson Central Terminal. A minimum of 30 separate unipolar recordings of 30-seconds were collected to ensure adequate LA coverage.

The wavefront propagation maps created at a PI of 600 and 250 ms were reviewed to identify the presence of pivot points and their relationship with the underlying voltage and RDCV slowing sites. Pivot points were defined as sites that demonstrated a change in wavefront propagation of ≥90°.

### Impact of pulmonary vein isolation on conduction velocity dynamics curves and distribution of rate-dependent conduction velocity slowing sites

A subgroup of patients had CV maps created post-PVI utilizing the same methodology used pre-PVI. The CV dynamic curves obtained at the three pre-defined voltage zones post-PVI were compared to those obtained pre-PVI. The CV measurements were also used to compare the anatomical distribution and proportion of RDCV slowing sites pre- and post-PVI by co-registering the maps. Rate-dependent CV slowing sites were deemed to anatomically co-locate if the RDCV slowing sites on the two maps were within a 3 mm geodesic distance from each other.

### Computational modelling studies

#### Impact of slower conduction velocity and enhanced fibrosis on rotational activity burden

In a subset of 10 cases, personalized anatomical models with labelled PVs and fibres were produced and analysed using an automated algorithm using python.^9,10^ The impact of slower CV (*conductivity models*) and enhanced fibrosis (*percolation models*) on rotational activity burden was assessed with the models calibrated to a PI of 600 ms. The two models were assessed individually and in combination. Post-ablation simulations were produced using CARP simulator in each model, where PVI was applied to each model 5 s after AF initiation. Wavefront propagation patterns were assessed using previously published phase mapping algorithms^11^ to calculate the number of rotational activities on the six-segment anatomical model of the LA. Rotational activities were defined as specific regions or areas where a spiral wave is observed causing disorder in the LA. All simulations used the Courtemanche *et al.*^12^ ionic cell model with AF electrical remodelling, with the monodomain model for tissue propagation. For healthy non-fibrotic tissue, the longitudinal conductivity was 0.4 S/m and the transverse conductivity was 0.107 S/m. For each case, fibrosis was included in the model depending on the BV value at PIs of 600 ms. Each triangular element of the mesh was assigned a BV value through interpolation. Fibrotic areas were defined as areas with a BV <0.5 mV.^13^ For the conductivity models, fibrotic areas were assigned low CVs: longitudinal conductivity of 0.1 S/m and transverse conductivity of 0.025 S/m. These models will allow the evaluation of the impact enhanced slow CV has on rotational activity burden. Percolation represents a type of complete replacement fibrosis in which myocytes are replaced by scar and removed from the mesh. The percolation models thereby allow the evaluation of the impact enhanced scar has on rotational activity burden.

#### Impact of slower conduction velocity and functional remodelling seen on 250 ms maps on rotational activity burden

Using the same 10 cases analysed in the previous section, for each case, fibrosis was included in the model depending on the BV value at PIs of 600 and 250 ms. Each triangular element of the mesh was assigned a BV value through interpolation. The tissue conductivities were calibrated at PIs of 600 and 250 ms to each of the distinct CV maps to construct a set of possible models. The aim was to determine whether the greater proportion of slower CV sites and presence of functional remodelling seen on a PI of 250 ms maps compared with a PI of 600 ms maps impacted the rotational activity burden.

### Statistical analyses

Statistical analyses were performed using SPSS (IBM SPSS Statistics, version 25, IBM Corp., NY, USA) (see Supplementary material online, Methods).

## Results

### Baseline characteristics

Sixty-two patients were included (see Supplementary material online, *Table S1*). No complications were encountered. Two patients were excluded from the study, as DCCV did not restore SR leaving 60 patients (see Supplementary material online, *Figure S1*).

### Conduction velocity and conduction velocity dynamics and the relationship with voltage

A total of 2 768 400 LAT and voltage points were analysed (46 140 ± 5689 points per patient, 7690 ± 1211 points per map). The average CV obtained at a PI of 600 ms was 1.22 ± 0.20 m/s. The CV obtained across the three voltage zones at this PI was significantly different [1.59 ± 0.13 ms nLVZs (≥0.5 mV), 1.05 ± 0.26 ms LVZs (0.2–0.49 mV), and 0.58 ± 0.37 ms vLVZs (<0.2 mV); *P* < 0.001]. There was a positive correlation between the mean CV for a patient and mean BV (*r*_s_ = 0.91, *P* < 0.001; Supplementary material online, *Figure S2A*) and proportion of nLVZs (*r*_s_ = 0.86, *P* < 0.001; Supplementary material online, *Figure S2B*).

**Figure 1:**
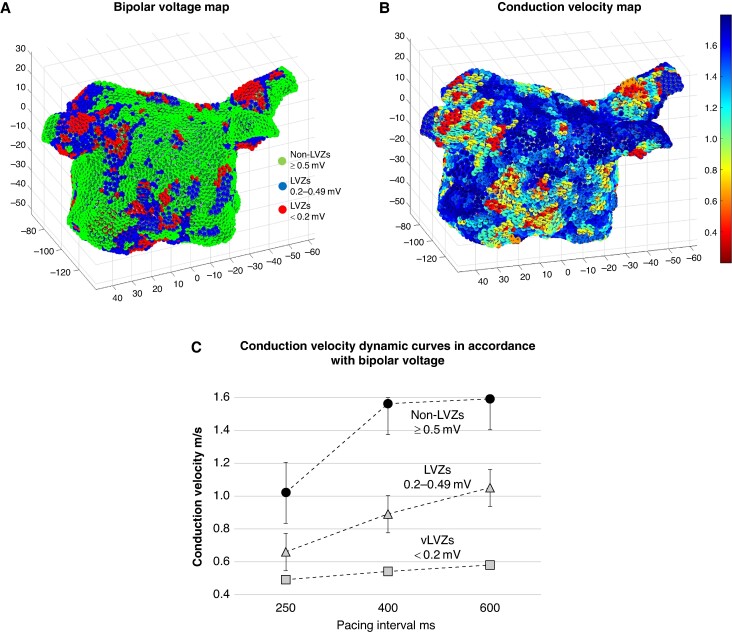
(*A*) BV map in SR in a posterior–anterior (PA) view [green ≥0.5 mV (nLVZs), blue 0.20–0.49 mV (LVZs), and red <0.20 mV (vLVZs)]. (*B*) CV map and (*C)* CV dynamic curves in accordance with BV (black circle ≥0.5 mV, light grey triangle 0.2–0.49 Mv, and light grey square <0.2 mV). BV, bipolar voltage; CV, conduction velocity; LVZs, low-voltage zones; nLVZs, non-low-voltage zones; SR, sinus rhythm; vLVZs, very low–voltage zones.

The CV dynamic curves obtained at the three PIs were different across the three pre-defined voltage zones. Very low–voltage zones (<0.2 mV) demonstrated flat curves with minimal change in CV with rate. In contrast, LVZs (0.2–0.49 mV) showed broader CV dynamic curves with a progressive change in CV with rate. Non-LVZs (≥0.5 mV) demonstrated steeper CV dynamic curves with a minimal change in CV between 600 and 400 ms, whilst the CV change between 400 and 250 ms was greater (*Table 1* and *Figure 1A–C*).

**Table 1:** The CV changes across the three PIs stratified in accordance with the three pre-defined voltage zones

	Non-LVZs( ≥ 0.50mV)	LVZs (0.2–0.49 mV)	vLVZs (<0.2 mV)	*P*-value
CV at 600 ms, m/s mean ± SD	1.59 ± 0.13	1.05 ± 0.26	0.58 ± 0.37	<0.001
CV change, m/s mean ± SD				
600–400 ms PIs	0.03 ± 0.02	0.16 ± 0.09	0.04 ± 0.01	<0.001
400–250 ms PIs	0.54 ± 1.10	0.23 ± 1.10	0.05 ± 0.02	<0.001
RDCV slowing sites, % mean ± SD	23.8 ± 15.0	76.2 ± 15.5	0	<0.001
Change in CV at RDCV slowing sites, m/s mean ± SD				
600–400 ms PIs	0.07 ± 0.02	0.17 ± 0.05		<0.001
400–250 ms PIs	0.65 ± 0.09	0.30 ± 0.07		<0.001

CV, conduction velocity; LVZs, low-voltage zones; nLVZs, non-low-voltage zones; PIs, pacing intervals; RDCV, rate-dependent conduction velocity; vLVZs, very low–voltage zones.

Rate-dependent CV slowing sites were identified in all patients, with an average of 2.8 ± 1.2 RDCV slowing sites per patient with a total of 168 RDCV slowing sites identified. Correlating the RDCV sites with the underlying voltage at PIs of 600 ms, RDCV slowing sites were predominantly seen in LVZs (0.2–0.49 mV) (128/168, 76.2%). No RDCV slowing sites were demonstrated in vLVZs (<0.2), and the remaining 40 (23.8%) RDCV slowing sites were seen in nLVZs (≥0.5 mV). There was a positive correlation between proportion of the LA area occupied by LVZs (0.2–0.49 mV) and the number of RDCV slowing sites identified (*r*_s_ = 0.89). Rate-dependent CV slowing sites mapped to nLVZs (≥0.5 mV) demonstrated steep CV dynamic curves, whilst those mapped to LVZs (0.2–0.49 mV) demonstrated broad curves (*Table 1*).

### Fixed and functional remodelling

**Figure 2:**
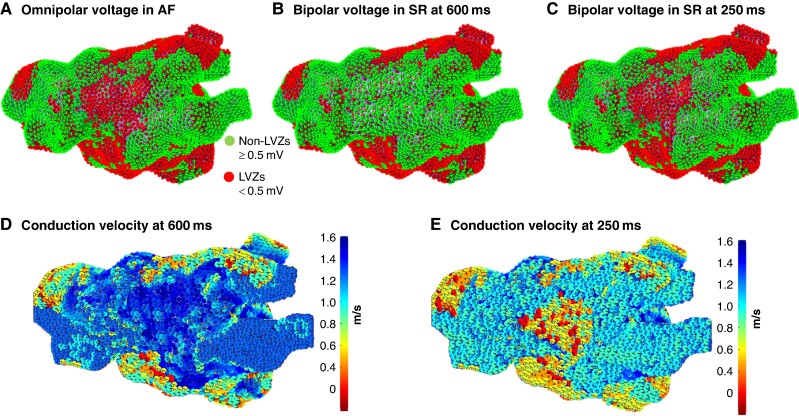
(*A*) AF OV map in an anterior–posterior (AP) view. (*B*) SR BV 600 ms map. (*C*) SR BV 250 ms map. AF OV and SR BV 250 ms maps show a LVZ anteriorly not shown on the SR BV 600 ms map. (*D*) CV map at a PI of 600 ms that demonstrates normal CV anteriorly. (*E*) CV map at a PI of 250 ms that demonstrates slow CV anteriorly. AF OV, atrial fibrillation omnipolar voltage; CV, conduction velocity; LVZ, low-voltage zone; PI, pacing interval; SR BV, sinus rhythm bipolar voltage.

In the 60 patients, 180 voltage maps were reviewed (60 AF OV, 60 SR BV 600 ms, and 60 SR BV 250 ms maps). Across an average of 7618.0 ± 1308.9 points per map, there was a significant difference in the average voltage between AF OV and SR BV 600 ms maps (0.42 ± 0.15 mV AF OV vs. 0.60 ± 0.20 mV SR BV 600 ms; *P* = 0.001). These findings were also consistent with regard to the proportion of the LA area occupied by LVZs, and the voltage at co-registered points was significantly different (*Table 2*). The AF OV maps identified an additional 144 LVZs (2.4 ± 1.8 LVZs per patient) (*Figure 2A–E*). Out of these, 128 (88.9%) LVZs were identified on SR BV 250 ms maps. The voltage parameters comparing AF OV maps and SR BV 250 ms maps were not significantly different (*Table 2*). Utilizing co-registered points, the voltage at the LVZs identified on the AF OV maps and not SR BV 600 ms maps was significantly different between SR BV 600 ms maps and SR BV 250 ms maps (0.63 ± 0.21 mV BV SR 600 ms vs. 0.34 ± 0.18 mV BV SR 250 ms; *P* = 0.002).

**Table 2:** Differences in voltage parameters between the AF OV, SR BV 600 ms, and SR BV 250 ms maps

Voltage parameters	AF OV *n* = 60	SR BV 600 *n* = 60	BV SR 250 *n* = 60	*P*-value AF OV vs. SR BV 600	*P*-value AF OV vs. SR BV 250	*P*-value SR BV 600 vs. 250
Voltage, mV mean ± SD	0.42 ± 0.15	0.60 ± 0.20	0.38 ± 0.12	0.001		0.001
Proportion of voltage points assigned low voltage, % mean ± SD	64.3 ± 12.9	51.4 ± 10.3	59.3 ± 12.1	0.002	0.09	0.01
Proportion of LA area occupied by LVZs, % mean ± SD	43.4 ± 10.3	30.2 ± 14.1	40.1 ± 11.5	0.001	0.12	0.01
Voltage difference at co-registered points between AF OV maps and SR BV maps, mV mean ± SD	0.16 ± 0.03 (AF OV vs. SR BV 600) 0.05 ± 0.04 (AF OV vs. SR BV 250)		0.13 ± 0.1 (SR BV 600 vs. SR BV 250)	0.02	0.10	0.03

AF OV, atrial fibrillation omnipolar voltage; LA, left atrial; SR BV, sinus rhythm bipolar voltage.

When reviewing the CVs at LVZ on AF and SR BV 250 ms maps but not on SR BV 600 ms, the CVs were significantly lower, more so than accounted by changes secondary to rate, on the SR BV 250 ms compared with the SR BV 600 ms maps (0.82 ± 0.18 m/s vs.1.54 ± 0.22 m/s; *P* < 0.001). Further to this, out of the 40 RDCV slowing sites that were mapped to nLVZs, 29 (72.5%) of these were mapped to LVZs on the SR BV 250 ms maps.

Anatomically, functional remodelling sites more frequently involved the anterior (45/128, 35.2%), posterior (37/128, 28.9%), and inferior (31/128, 24.2%) wall. The proportion of the LA area occupied by functional remodelling was significantly greater in patients with a lower proportion of fixed LA remodelling <50% compared with patients with fixed LA remodelling of ≥50% (34.4 ± 15.2% vs. 18.2 ± 16.2%; *P* = 0.002).

### Pivot points

**Figure 3:**
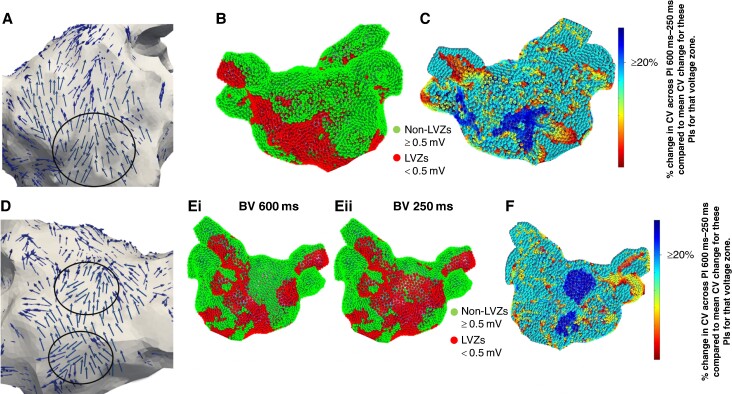
(*A*) Wavefront propagation map in an anterior–posterior (AP) view where the arrows highlight the wavefront propagation along the anterior wall with a pivot point (black circle). The pivot point co-locates to a LVZ (red) identified on (*B*) SR BV 600 ms map and a RDCV slowing site (blue) on the (*C*) CV map. The CV map is created by taking the reduction in CV between 600 and 250 ms at a site and then determining the percentage reduction when compared with the average reduction in CV between 600 and 250 ms for that voltage zone. Blue highlights sites with a CV change of ≥20%, i.e. RDCV slowing site. (*D*) Wavefront propagation map in a PA view where the arrows highlight the wavefront propagation along the posterior wall with two pivot points (black circles). The pivot point mapped to the low posterior wall co-located to a LVZ (red) on the (*Ei*) SR BV 600 ms map and (*Eii*) SR BV 250 ms map. The other pivot point mapped to the mid-posterior wall co-located to a nLVZ (green) on the SR BV 600 ms map but a LVZ on the SR BV 250 ms map. Both pivot points also co-located to a RDCV slowing site (blue) on the (*F*) CV map. CV, conduction velocity; LVZ, low-voltage zone; RDCV, rate-dependent conduction velocity; SR BV, sinus rhythm bipolar voltage.

A total of 183 pivot points (3.05 ± 1.1 pivot points per patient) were identified on the 600 ms PI wavefront propagation maps. Out of these pivot points, 161 (88.0%) and 151 (82.5%) co-located to sites of LVZs and RDCV slowing, respectively (representative *Figure 3A–F*). All these pivot points were mapped to RDCV slowing sites that were mapped to LVZs (0.2–0.49 mV). An additional 42 pivot points were identified on the 250 ms PI wavefront propagation maps. Out of these, 37 (88.1%) co-located to sites of LVZs on the SR BV 250 ms maps but nLVZs on the SR BV 600 ms maps.

Reviewing the wavefront propagation maps at the functional remodelling sites, only 37 out of the 128 (28.9%) LVZs on the SR BV 250 ms maps co-located to sites with a change in wavefront direction. The remaining LVZs seen on the SR BV 250 ms map but not on the SR BV 600 ms maps, i.e. functional remodelling sites, showed no change in wavefront directionality on the 250 ms PI wavefront propagation maps compared with the 600 ms PI wavefront propagation maps. In the 37 LVZs that co-located with a change in wavefront propagation, the change in wavefront direction only occupied on average 24.5 ± 14.5% of the area of the LVZ.

The CV dynamic changes seen with voltage, the correlation between AF OV maps and SR BV 250 maps, and the increase in number of pivot points on SR BV 250 ms maps compared with SR BV 600 ms maps were consistent across all patients regardless of their underlying baseline characteristics.

### Impact of pulmonary vein isolation on conduction velocity dynamics curves and distribution of rate-dependent conduction velocity slowing sites

Forty patients had CV maps created post-PVI. When comparing the CV dynamic curves post-PVI to those pre-PVI, there was no significant change in the CV measurements across the three PIs for the three pre-defined voltage zones (see Supplementary material online, *Table S2*). As a result, the CV dynamic curves maintained the same pattern across the three pre-defined voltage zones as per the CV dynamic curves pre-PVI with steep curves seen at nLVZs (≥0.5 mV), broad curves seen at LVZs (0.2–0.49 mV), and flat curves seen at vLVZs (<0.2 mV).

Post-PVI, 100 RDCV slowing sites were identified, with an average of 2.5 ± 1.1 RDCV slowing sites per patient. All these RDCV slowing sites anatomically co-located to RDV slowing sites identified pre-PVI. In the 40 patients, 12 RDCV slowing sites were identified pre-PVI that were not seen post-PVI of which 10 (83.3%) were mapped close to the WACA lines.

### Computation modelling findings

#### Impact of slower conduction velocity and enhanced fibrosis on rotational activity burden

**Figure 4:**
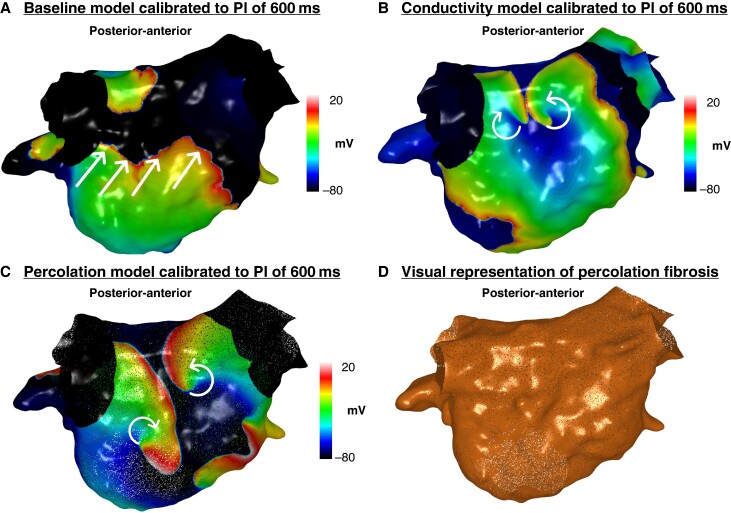
Simulated propagation patterns as transmembrane potential map in a posterior–anterior view generated from the (*A*) baseline personalized model calibrated to a PI of 600 ms, (*B*) conductivity model calibrated to a PI of 600 ms, and (*C*) percolation model calibrated to a PI of 600 ms. The straight lines indicate the propagation direction of a non-rotational wavefront. The curved arrows indicate a rotational wavefront propagation. Both conductivity and percolation models demonstrate rotational activity on the posterior wall, whilst this was not seen on the baseline model. (*D*) Visual representation of percolation fibrosis. PI, pacing interval.

The total number of rotational activities and number of rotational activities per patient were greater on the conductivity models (*n* = 35, 3.5 ± 2.1 per patient baseline models vs. *n* = 49, 4.9 ± 2.3 per patient conductivity model; *P* = 0.04) and percolation (*n* = 35, 3.5 ± 2.1 per patient baseline models vs. *n* = 58, 5.8 ± 4.3 per patient percolation models; *P* = 0.04) models compared with the baseline models calibrated to a PI of 600 ms (*Figure 4A–D*). There was also a significant increase in the total burden and per patient burden of rotational activity mapped to LVZs in both the conductivity (*n* = 16, 1.6 ± 1.5 per patient, baseline model vs. *n* = 35, 3.5 ± 1.6 per patient conductivity model; *P* = 0.009) and percolation (*n* = 16, 1.6 ± 1.5 per patient, baseline model vs. *n* = 45, 5.6 ± 2.9 per patient percolation model; *P* = 0.009) models compared with the baseline models. The percolation models included eight models as AF termination occurred in two models.

#### Impact of slower conduction velocity and functional remodelling seen on 250 ms maps on rotational activity burden

The total number of rotational activities and number of rotational activities per patient were greater on the models calibrated to a PI of 250 ms than 600 ms (*Table 3*). A larger proportion of rotational activities were mapped to LVZs on models calibrated to 250 ms compared with 600 ms (51/52, 98.1% 250 ms vs. 16/35, 45.7% 600 ms; *P* < 0.001).

**Table 3:** Differences in the burden of rotational activities mapped to the models calibrated to PIs of 600 and 250 ms

	Models calibrated to a PI of 600 ms, *n* = 10	Models calibrated to a PI of 250 ms, *n* = 10	*P*-value
Total number of rotational activities, *n*	35	52	
Number of rotational activities mapped per patient, mean ± SD	3.5 ± 2.1	5.2 ± 1.9	0.04
Total number of rotational activities mapped to LVZs, *n*	16	51	
Number of rotational activities mapped to LVZs per patient, mean ± SD	1.6 ± 1.5	5.1 ± 1.8	<0.001
Anatomical distribution of rotational activities mapped to LVZs per patient, *n* mean ± SD			
Anterior	0.7 ± 1.0	1.8 ± 1.3	0.04
Inferior	0	1.0 ± 0.9	0.002
Posterior	0.5 ± 0.7	1.3 ± 1.0	0.03
Roof	0.1 ± 0.6	0.5 ± 0.7	0.22
Lateral	0.3 ± 0.6	0.5 ± 0.7	0.22
Septum	0	0	

PI, pacing interval; LVZs, low-voltage zones.

Out of the 35 rotational activities identified on the models calibrated to a PI of 600 ms, many were seen on models calibrated to a PI of 250 ms (24/35, 68.6%) and the ones that were not were predominantly mapped to nLVZs on both models (10/11, 90.9%). The models calibrated to a PI of 250 ms demonstrated 17 additional rotational activities (2.0 ± 1.8 additional activities per patient). All these activities were mapped to LVZs on the SR BV 250 ms maps but nLVZ on the SR BV 600 ms. When only reviewing the activities mapped to LVZs representing either site of fixed or functional remodelling, there were again a significantly higher number of activities identified on the models calibrated to a PI of 250 ms compared with a PI of 600 ms (*Table 3*).

**Figure 5:**
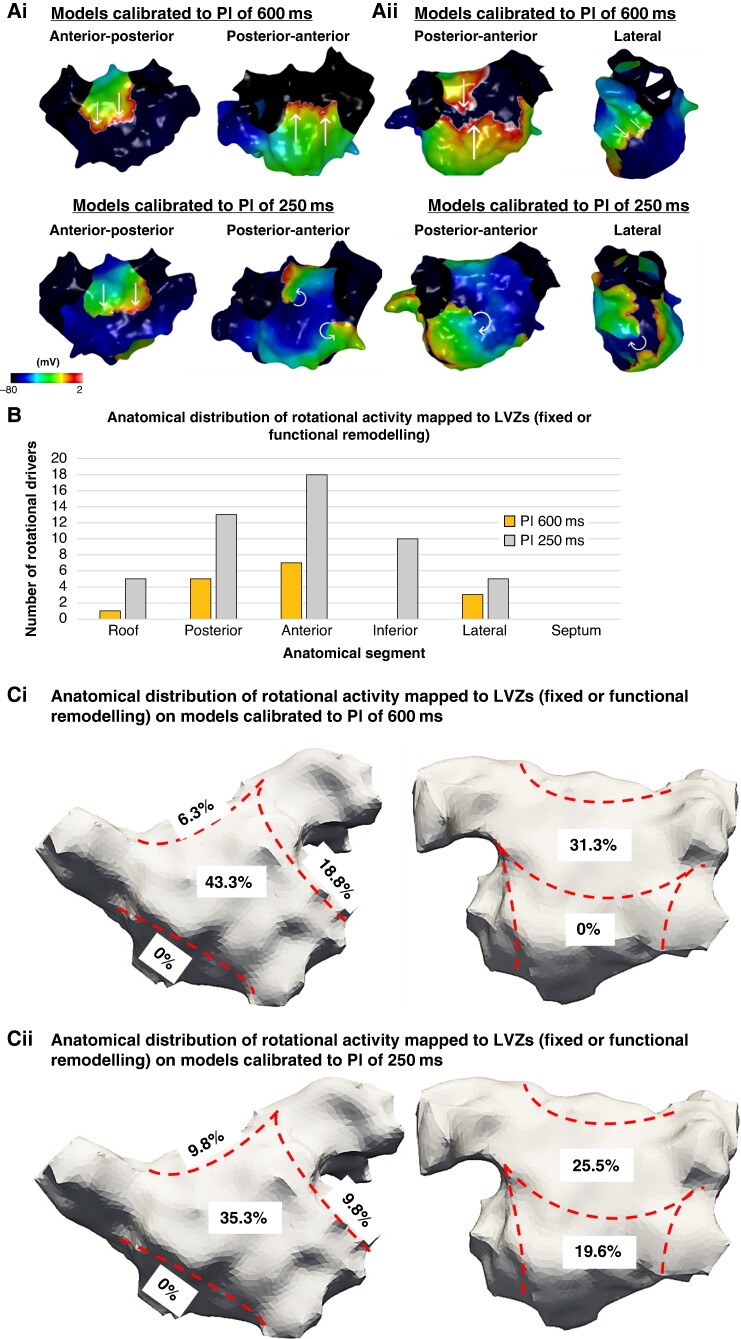
(*Ai–ii*) Simulated propagation patterns as transmembrane potential for two personalized models calibrated to a PI of 600 and 250 ms post-PVI simulations. The straight lines indicate the propagation direction of a non-rotational wavefront. The curved arrows indicate a rotational wavefront propagation. (*Ai*) Maps in an anterior–posterior (AP) and posterior–anterior (PA) view. The PA maps showed two rotational activities on the models calibrated to a PI of 250 ms which was not present on the models calibrated to a PI of 600 ms. (*Aii*) Maps in a PA and lateral view. The PA and lateral views demonstrate a rotational activity on the models calibrated to a PI of 250 ms which was not present on the models calibrated to a PI of 600 ms. (*B*) The histogram demonstrates the anatomical distribution of the rotational activities mapped to LVZs (fixed or functional remodelling) with an increase in the number of rotational activities identified on models calibrated to a PI of 250 ms compared with the models calibrated to a PI of 600 ms. This was particularly evident on the anterior and inferior wall. (*Ci–ii*) The proportion of rotational activities mapped to LVZs in accordance with anatomical segments on models calibrated to (*i*) a PI of 600 ms and (*ii*) a PI of 250 ms. LVZ, low-voltage zone; PI, pacing interval; PVI, pulmonary vein isolation.

The anatomical distribution of the activities was similar across both models with a majority being mapped to the anterior and posterior wall (*Figure 5A–C*). All anatomical segments except septum harboured a higher number of activities on the models calibrated to a PI of 250 ms and consistent with the anatomical distribution of functional remodelling.

## Discussion

This is the first study to have extensively investigated the interaction between structural remodelling and electrophysiological properties with regard to CV dynamics. This study has established the importance of areas with moderate fixed remodelling, areas with disproportionate CV slowing at shorter CLs (RDCV slowing), and functional remodelling evident at shorter CLs as part of a potential new paradigm in contrast to the conventional target of fixed remodelling.

**The key findings were as follows:**

1. The extent of the structural remodelling in terms of the voltage determined the CVs and CV dynamics. LVZs (0.2–0.49 mV) and not LVZs (<0.2 mV) showed dynamic changes in CV across the whole range of CLs studied and were most likely to give rise to CV heterogeneity.
2. A novel marker for CV slowing at shorter CLs termed RDCV slowing identified areas with enhanced CV heterogeneity which were found mostly in LVZs (0.2–0.49 mV) and not LVZs (<0.2 mV).
3. In addition to LVZs representing fixed remodelling evident on SR BV 600 ms maps, there were additional LVZs evident on SR BV 250 ms maps representing functional remodelling. Areas of both fixed and functional remodelling demonstrated abnormal CV dynamics together with pivot points during pacing in SR.
4. AF OV maps demonstrated additional LVZs not seen on SR BV 600 ms, but a majority were seen on SR BV 250 ms. These data suggest that LVZs on OV maps in AF likely represent a combination of fixed remodelling evident at slower pacing rates, but also functional remodelling only evident at higher pacing rates.
5. Computational modelling studies demonstrate that when calibrating to slower CVs and enhanced fibrosis, there is an increase in rotational activity burden in AF. These studies also demonstrated that rotational activity was drawn predominantly to LVZs but also areas of functional remodelling identified only at CLs of 250 ms, emphasizing their mechanistic importance in re-entry formation.

### The relationship of conduction velocity and conduction velocity dynamics with voltage

As shown in previous studies, CV is lower in areas of scar compared with healthy tissue.^4,5^ However, different levels of structural remodelling not only impact the CV at 600 ms but also influence the CV dynamics: (i) nLVZs (≥0.5 mV) curves were ‘steep’ meaning little change from 600 to 400 ms and then a sharp fall in CV at 250 ms, (ii) LVZs (0.2–0.49 mV) curves were ‘broad’ with a progressive decrease in CV across shorter CLs, and (iii) vLVZs (<0.2 mV) curves were flat with slow CV even at 600 ms and little change at shorter CLs. Why these voltages zones demonstrated different CV dynamic curves remains unclear. It is plausible that LVZs (0.2–0.49 mV) are healthy enough to conduct relatively normally at longer CLs but respond quickly to shortening of CL, whilst the level of disease in vLVZs (<0.2 mV) results in no conduction reserve with poor conduction even at lower CLs and thereby a minimal change in CV with rate. Studies have also shown that the type of histological changes has a direct impact on CV whereby dense diffuse fibrosis with short fibrotic strands only marginally affect conduction curves but long fibrotic strands promote prominent increases in activation delay.^14^ Thin interstitial collagen strands lower CV compared with thick interstitial collagen strands.^15^ Fibroblast–myocyte coupling has also shown to have competing effects on the CV.^16^ Further to this, structural remodelling results in fibroblast–myocyte coupling which has shown to impact CV.^16^ Replacement fibrosis also impacts action potential duration^17^ and promotes CV heterogeneity.^14^ Relative differences in these contributing factors could account for the differing CV dynamics in the different levels of LVZs.

### Rate-dependent conduction velocity slowing sites

The differences in CV dynamics also resulted in differences in the distribution of sites of enhanced CV heterogeneity, i.e. RDCV slowing sites. These occurred predominantly in LVZs (0.2–0.49 mV). Rate-dependent CV slowing sites have previously been shown to correlate with sites of re-entry activity in AF and AT.^4,5^ In this study, RDCV slowing sites co-located to pivot points during paced maps in SR, further highlighting that CV heterogeneity impacts wavefront propagation in a manner promoting re-entry.

Even though RDCV slowing sites were also mapped to nLVZs, the CV dynamic curves for these sites were steep in contrast to the broad curves seen for these sites when mapped to LVZs (0.2–0.49 mV). Broad CV dynamic curves have shown to have alter activation vector and promote arcing with accelerated rates which may reflect rate-dependent conduction block in certain directions^6^ that can promote re-entry.^18^ This is consistent with the findings in this study, whereby pivot points only co-located to RDCV slowing sites mapped to LVZs (0.2–0.49 mV). Further to this, a large proportion of RDCV slowing sites mapped to nLVZs in SR at a PI of 600 ms demonstrated a drop in the peak-to-peak voltage at a PI of 250 ms, which suggests that this tissue is abnormal and demonstrates sites of functional remodelling. In this study, CV was assessed using an automated algorithm and RDCV slowing sites were used as a marker for CV heterogeneity. Other studies have utilized features of conduction block as defined by differences in activation times to determine degree of conduction inhomogeneity.^19^ However, this has been performed using epicardial mapping only, and thereby, whether the findings are applicable to the endocardium is unclear. Further to this, the spatial relationship to re-entry activity in AT and AF has not been shown using this methodology, whilst this has been demonstrated using the methodology applied in this study.^4,5^

### Fixed and functional remodelling

There was a significant drop in peak-to-peak voltage with rate when comparing SR BV 600 ms with SR BV 250 ms maps. There were widespread LVZs on SR BV 250 ms maps not seen on SR BV 600 ms maps suggestive of functional remodelling. Even though AF OV maps have shown to better correlate with BV maps in SR compared with BV maps in AF,^8^ OV maps in AF still demonstrate LVZs not identified on SR BV 600 ms maps. However, the voltage maps created in SR at a higher pacing rate were very similar to the AF OV maps. Bipolar voltage maps at faster pacing rates may represent areas of functional remodelling. Although many mechanisms have been proposed for LVZs identified in AF but not in SR, these findings point to functional remodelling as a likely cause.

Previous studies have demonstrated the functional nature of atrial substrate.^20,21^ However, this is the first study that has evaluated the impact of functional remodelling on enhanced CV heterogeneity sites and pivot points in SR in humans. Functional remodelling sites resulted in enhanced CV heterogeneity sites, i.e. RDCV slowing sites which have been shown to co-locate to re-entry activity in AF^5^ and AT.^4^ Further to this, functional remodelling sites also resulted in an increase in pivot points which have been shown to be a precursor to re-entry activity.

It is hard to establish whether the rate-dependent drop in voltage is a cause or effect of rate-dependent conduction delay. A previous study revealed sites of conduction delay with atrial pacing. However, these sites did not correlate with sites of drop in voltage suggesting that conduction delay does not consistently cause a drop in voltage.^20^ Further to this, in another study the drop in BV with atrial rate was more remarkable and out of proportion to the smaller drop in CV seen.^21^ Regardless of the specifics of this relationship, both CV slowing and reduction in voltage predispose to re-entry formation and are potential contributors to the pathophysiology of AF.

Patients with <50% fixed scar had a greater proportion of functional remodelling, and thereby, it is plausible that there is a continuum whereby functional remodelling may be partly a precursor to the development of fixed remodelling. Further to this, pivot points were mapped to both sites of fixed and functional remodelling, highlighting the impact both processes have on wavefront propagation and the potential for re-entry formation.

### Computational modelling studies

The conductivity and percolation models demonstrated an increase in rotational activity burden on the models calibrated to PIs of 600 ms emphasizing the mechanistic importance of slow CV and enhanced fibrosis on re-entry formation. The models calibrated to PIs of 250 ms which considered slower CVs and functional remodelling also demonstrated a greater rotational activity burden compared with the models calibrated to PIs of 600 ms. Furthermore, the additional rotational activities identified co-located to sites of functional remodelling evident only on the 250 ms maps. This demonstrates the likely mechanistic significant of CV heterogeneity and functional remodelling in promoting re-entry and rotational activity formation in AF and lends further weight to these as potential targets for ablation.

There is conflicting evidence for localized driver ablation as an ablation strategy for persistent AF, and currently, there are no recent randomized controlled trials that have shown superiority over PVI. The findings for STAR AF 3 trial are eagerly awaited. Our group has extensively evaluated the role of localized driver ablation in AF.^5,22–25^ Whilst these non-randomized controlled studies have shown high rates of freedom from AF/AT with localized driver ablation, more importantly these studies have demonstrated that localized drivers have a role in AF mechanisms. In this study, computational modelling was utilized to assess rotational activity burden and its relationship with fixed and functional remodelling and enhance CV heterogeneity. The focus of this part of the study was to gain further insight into the mechanistic role of these substrate characteristics in promoting rotational activity rather than evaluating the role of rotational driver ablation which needs to be further evaluated in randomized controlled trials.

### Clinical implications for substrate modification

Low-voltage zones’ substrate modification as an ablation strategy for persistent AF has shown to be associated with a higher freedom from atrial arrhythmia compared with a PVI only ablation strategy.^2,26^ Current substrate modification approaches have focused on fixed remodelling identified in SR.^2,27^ Non-invasive assessment of fixed remodelling has also been explored using cardiac magnetic resonance with variable accuracy.^28–30^ However, the findings in this study suggest that fixed remodelling is only part of the substrate modification picture. Low-voltage zones (0.2–0.49 mV) were the most common sites of RDCV slowing causing enhanced CV heterogeneity and were associated with pivot points during pacing. Sites of functional remodelling only evident at faster pacing rates or during AF were also associated with RDCV slowing and pivot points. Computational modelling suggested that both fixed and functional remodelling sites were critical for rotational activity in AF.

These data suggest that functional remodelling is at least as important as fixed remodelling in AF mechanisms and could be an important ablation target. Identification of functional remodelling sites requires voltage mapping at a pacing CL of 250 ms, although if this cannot be achieved without initiating AF, then an OV map in AF is very similar and could be used as a surrogate.

Conversely, studying CV to determine RDCV slowing sites may allow a conservative approach targeting less tissue at the expense of more time mapping. This is the first study to explore the relationship between fixed remodelling, functional remodelling, and CV dynamics in patients with AF and raises many questions about how atrial substrate is targeted, potentially offering a rationale to modify current methods and outlines potential new targets for ablation. These data suggest that there is enormous scope for modifying areas of fixed and functional remodelling beyond the current strategy for homogenizing areas of fixed remodelling. Currently, because of the lack of substantial evidence no advice is offered by the European Heart Rhythm Association/Heart Rhythm Society/Asia Pacific Heart Rhythm Society/Latin American Heart Rhythm Society expert consensus statement^31^ regarding substrate modification of LVZs as an ablation strategy for persistent AF. The findings from this study provide further evidence of the role in re-entry formation of both fixed and functional remodelling in persistent AF, which further strengthens the evidence for a substrate modification of LVZs ablation approach in persistent AF. Several ablation approaches have been explored beyond PVI for persistent AF including localized driver ablation which has shown conflicting evidence.^32^ It is well recognized that a better understanding of the pathophysiology of AF would aid in developing additional ablation strategies. This study provides further insight into the pathophysiology of AF and identifies additional potential ablation targets which should be further evaluated in randomized controlled trials.

### Limitations

Several refinements to current methods of substrate modification could be inferred from these data including a potential role for the novel target of RDCV slowing sites. The impact of these strategies requires further evaluation in randomized controlled studies.

In this study, we focused on using voltage assessment with endocardial contact mapping. Substrate assessment using this approach is well recognized and used as the gold standard for assessment of fibrosis and scar. Whilst several non-invasive approaches are being utilized to assess underlying substrate such as surface ECG parameters including *P* wave characteristics,^33,34^ the evidence for their role is still limited and they are still not recognized as a replacement to contact mapping for fibrosis and scar assessment in the atrium. The role of ECG parameters in identifying fixed and functional remodelling sites still needs further evaluation.

In this study, the focus was the endocardium because currently non-surgical catheter ablation approaches focus on the endocardium. Further to this, substrate modification of LVZs studies has focused on homogenizing LVZs in the endocardium. Thereby, the findings from this study are additive to our current ablation strategies for persistent AF making them clinically relevant. However, gaining a greater understanding of atrial epicardial substrate and its mechanistic role in AF is of great interest and can provide further insight to AF mechanisms and novel ablation strategies in AF.

## Conclusions

Although voltage mapping in SR can identify fixed atrial structural remodelling as LVZs, this is arguably a limited view of atrial remodelling. Areas of moderate fixed remodelling and areas of functional remodelling evident only at faster pacing rates were arguably more mechanistically important than areas of fixed scar and may represent targets for ablation. Low-voltage zones identified on OV maps in AF, correlated with areas of fixed remodelling and functional remodelling identified at shorter pacing CLs, suggesting that OV maps in AF may be a reasonable surrogate to identify these sites of remodelling. Conduction velocity and CV dynamics are impacted by both fixed and functional remodelling. Potential novel targets termed RDCV slowing sites which give rise to localized CV heterogeneity at shorter CLs were identified predominantly in areas of moderate fixed remodelling and functional remodelling. Further exploration of these findings is warranted in terms of mechanistic studies but also testing these parameters identified as novel targets in substrate modification for AF.

## Supplementary Material

euae239_Supplementary_Data

## Data Availability

The data underlying this article will be shared on reasonable request to the corresponding author.
