# Supplementary material for: The effect of fixed and functional remodelling on conduction velocity, wavefront propagation, and rotational activity formation in atrial fibrillation
Source: Europace. 2024 Sep 16;26(10):euae239. doi: 10.1093/europace/euae239 (PMC11481322; doi:10.1093/europace/euae239)
Supplement: euae239_Supplementary_Data [file euae239_supplementary_data.zip › Supplemental Figure Legends.docx]

**SUPPLEMENTAL FIGURE LEGENDS**

***Supplemental Figure 1-*** Study flow diagram

***Supplemental Figure 2A-B-*** Shows the relationship between the average CV at a PI of 600ms and ***A-*** Average bipolar voltage ***B-*** Proportion of non-LVZs.
