# Supplementary material for: The effect of fixed and functional remodelling on conduction velocity, wavefront propagation, and rotational activity formation in atrial fibrillation
Source: Europace. 2024 Sep 16;26(10):euae239. doi: 10.1093/europace/euae239 (PMC11481322; doi:10.1093/europace/euae239)
Supplement: euae239_Supplementary_Data [file euae239_supplementary_data.zip › Supplemental Methods final.docx]

**Supplemental Methods**

1. ***Scar assessment***

Ensite X (Abbott, Chicago, IL, USA) was used as the 3D mapping system. Left atrial (LA) anatomical maps were created using the HD-grid mapping catheter (Abbott, Chicago, IL, USA). All patients had a high-density omnipolar voltage (OV) map created in AF using the HD-grid catheter. Points that were ≥5mm from the geometry surface were filtered as not being in contact with the myocardium, and points acquired were respiratory gated to optimize the accuracy of anatomical localization. A minimum of 7000 OV points were collected per patient with the aim of ensuring adequate atrial coverage. The interpolation threshold was set to 5mm for surface color projection and points were collected aiming for complete LA coverage (i.e., with no area >5mm from a data point). Three voltage zones were defined, very low voltage zones (vLVZs) <0.2mV, LVZs [0.2-0.49mV] and non-LVZs (nLVZs) i.e., normal voltage zones ≥0.5mV. A decapolar catheter (Boston Scientific) was positioned in the coronary sinus (CS). The Tactiflex ablation catheter (Abbott) was used for ablation. Following the voltage map in AF and prior to ablation, patients underwent DCCV to SR. All patients had a repeat bipolar voltage (BV) map created in SR using the above protocol with atrial pacing at a pacing interval (PIs) of 600ms and 250ms.

For BV assessment, the bipole obtained from the electrodes along the spline of the HD-grid was used. The window of interest (WOI) was set to exclude the QRS complex. The number of atrial beats within the WOI was dependent on the AF cycle length and the ventricular rate in AF. The largest peak-to-peak voltage point was then determined from all the atrial beats in the WOI. Following this, all peak-to-peak voltage points identified within a 1mm sphere was then identified. The best duplicate algorithm was then applied whereby the timing annotations of all these points were analysed. The extreme outliers were excluded. The remaining points were analysed, and the average timing annotation was calculated. The point with the largest voltage with timing near the average timing was used for the final BV. For OV assessment, signals were obtained from three non-colinear electrodes that make up a clique. These are used to calculate the BV in all directions over 360 degrees. The BV with the largest bipolar peak-to-peak voltage is then used to compute a local virtual bipolar signal that represents the OV. Following this, all peak-to-peak voltage points identified within a 1mm sphere were identified. The voltage point with the highest OT certainty (numeric value ranging from 0-1 indicating how certain the calculated activation direction is) and largest voltage amplitude is then used for the final OV.

A decapolar catheter (Boston Scientific) was positioned in the coronary sinus (CS). The Tactiflex ablation catheter (Abbott) was used for ablation.

1. ***Conduction velocity methodology***

First LATs were obtained using omnipolar electrograms. Omnipolar technology in EnsiteX utilizes a traveling wave phenomenon and reviews electrical characteristics from 3 neighboring electrodes i.e., a clique to estimate wavefront directionality and thereby eliminate “bipolar blindness”. LATs were all reviewed manually to ensure accuracy in timing. Utilizing an automated algorithm developed and executed in Matlab (Mathworks, MA, USA) the left atrial anatomical mesh was recreated utilizing xyz coordinates for the vertices and faces that made up the geometry created in EnsiteX.

Second a local gradient method was used to calculate CV. To reduce the effects of spatial resolution when comparing results across cases, each electroanatomic mapping mesh was first re-meshed to an average resolution of 2mm using Meshtool software, and all calculations were performed on this mesh. The LAT measurements were interpolated to give a LAT for every node on the mesh. This was achieved using an inverse distance weighting interpolation through Meshtool software. The gradient of the interpolated activation times was then used to estimate CV for each element of the mesh through utilizing the xyz coordinates and determining the distance between each element.

1. ***Pivot points***

A novel wavefront tracking algorithm developed and executed in Matlab (Mathworks, MA, USA) was used offline to track wavefront propagation in SR. Unipolar recordings using the HD-grid catheter were collected by referencing to Wilson Central Terminal (WCT). A minimum of 30, 30-seconds unipolar recordings were collected to ensure adequate LA coverage.

All patients had 30-seconds unipolar recordings collected sequentially using the HD-grid catheter. To allow effective tracking of the wavefront using the algorithm, the sequential recordings overlapped, ensuring each segment had several overlapping recordings in each direction. Combining multiple overlapping unipolar recordings allowed a prediction of the wavefront propagation over an anatomical segment and over time to be determined through matching wavefront patterns obtained for each unipolar recording.

With each unipolar recording xyz coordinates were obtained for the electrode location to allow pairing of the electrodes to their neighboring electrodes and allow electrode position on the geometry to be determined. Electrodes were paired to their neighboring electrodes through comparing geodesic distance between electrodes.

The unipolar electrograms were then reviewed. Firstly, ventricular far field signals were filtered. Atrial signals were then annotated. Filtering of far field ventricular and atrial signals were evaluated manually to ensure accurate exclusion of far field signals and atrial signal annotation. Inaccurate atrial annotations due to noise or fractionated signals were excluded from the analysis. The atrial activations were then compared amongst these electrodes to track the wavefront propagation. The fixed atrial pacing enabled establishment of predicted wavefront propagation. The wavefront propagation maps created at PI of 600ms and 250ms were reviewed to identify the presence of pivot points. Pivot points were defined as sites that demonstrated a change in wavefront propagation of ≥90 degrees. The relationship between sites of pivot points and the underlying voltage and RDCV slowing sites were determined.

***iv) Statistical analyses***

Statistical analyses were performed using SPSS (IBM SPSS Statistics, Version 25 IBM Corp, NY, USA). Continuous variables are displayed as mean ± standard deviation (SD) or median (range). Categorical variables are presented as numbers and percentages. The Student T-test or Mann-Whitney U test was used for comparison of continuous variables. Fisher’s exact test was used for comparison of categorical variables. Spearman rank correlation coefficient was determined to assess the relationship between mean BV and proportion of nLVZs and mean CV. Spearman rank correlation coefficient was determined to assess the relationship between proportion of LA area occupied by LVZs [0.2-49mV] and the number of RDCV slowing sites. P-value of <0.05 was deemed significant.
