# Supplementary material for: The effect of fixed and functional remodelling on conduction velocity, wavefront propagation, and rotational activity formation in atrial fibrillation
Source: Europace. 2024 Sep 16;26(10):euae239. doi: 10.1093/europace/euae239 (PMC11481322; doi:10.1093/europace/euae239)
Supplement: euae239_Supplementary_Data [file euae239_supplementary_data.zip › Supplemental Table 1.docx]

***Supplemental Table 1-*** *Baseline characteristics*

| **Baseline characteristics** | **Cohort n=62** |
| --- | --- |
| Age yrs. mean ± SD | 60.6±11.0 |
| Male n (%) | 55 (88.7) |
| Diabetes mellitus n (%) | 6 (9.7) |
| Hypertension n (%) | 22 (35.5) |
| TIA/CVA^Ƭ^ n (%) | 7 (11.3) |
| Ischaemic heart disease n (%) | 8 (12.9) |
| Cardiac surgery n (%) | 1 (1.6) |
| Cardiomyopathy n (%) | 29 (46.8) |
| BMI kg/m^2^ n (%)  20-30  31-40  >40 | 38 (61.3)  20 (32.3)  4 (6.5) |
| Obstructive sleep apnoea n (%) | 9 (14.5) |
| Left ventricular EF^Ŧ^ ≥ 55% n (%) | 29 (46.8) |
| LA size mm n (%)  30-40  41-50  >50 | 21 (33.9)  33 (53.2)  8 (12.9) |
| AF duration months, mean ± SD | 19.1±10.1 |
| Previous AT ablation n (%)  Cavo-tricuspid isthmus-dependent flutter | 4 (6.5) |
| Current anti-arrhythmic or rate-controlling strategy  Beta-blockers including Sotalol  Amiodarone  Flecainide  Calcium channel blocker  Digoxin | 53 (85.5)  27 (43.5)  7 (11.3)  2 (3.2)  8 (12.9) |
| Current anticoagulation strategy  Warfarin  Direct oral anticoagulants | 0 (0.0)  62 (100.0) |

^Ƭ^TIA/CVA- Transient ischaemic attack/Cerebrovascular accident

^Ŧ^EF- Ejection fraction
