# Supplementary material for: The effect of fixed and functional remodelling on conduction velocity, wavefront propagation, and rotational activity formation in atrial fibrillation
Source: Europace. 2024 Sep 16;26(10):euae239. doi: 10.1093/europace/euae239 (PMC11481322; doi:10.1093/europace/euae239)
Supplement: euae239_Supplementary_Data [file euae239_supplementary_data.zip › Supplemental Table 2.docx]

***Supplemental Table 2-*** *Compares the CV changes across the 3 pacing intervals stratified in accordance with the 3 pre-defined voltage zones pre- and post-PVI.*

|  | Non-LVZs^Ƭ^ ≥0.50mV  Pre-PVI Post-PVI | LVZs [0.2-0.49mV]  Pre-PVI Post-PVI | vLVZs [<0.2mV]  Pre-PVI Post-PVI |
| --- | --- | --- | --- |
| CV^Ŧ^ at 600ms, m/s  mean ± SD, p-value | 1.59±0.13 1.57±0.14  p=0.65 | 1.05±0.26 1.06±0.26  p=0.56 | 0.58±0.37 0.57±0.36  p=0.59 |
| CV change, m/s  mean ± SD  600-400ms PIs^δ^  p-value  400-250ms PIs  p-value | 0.03±0.02 0.03±0.02  p=0.89  0.54±1.10 0.55±1.12  p=0.53 | 0.16±0.09 0.15±0.10  0.23±1.10 0.24±1.12  p=0.73 | 0.04±0.01 0.04±0.01  0.05±0.02 0.05±0.03  p=0.74 |
| RDCV^\|\|^ slowing sites, % mean ± SD, p-value  ^Ƭ^LVZ- Low voltage zone  ^Ŧ^CV- Conduction velocity  ^δ^PI- Pacing interval  ^\|\|^RDCV- Rate dependent conduction velocity | 24.3±15.0 25.2±16.1  p=0.89 | 75.7±15.5 74.8±14.3  p=0.86 | 0 0 |
