# Supplementary figures and images for: The effect of fixed and functional remodelling on conduction velocity, wavefront propagation, and rotational activity formation in atrial fibrillation
Source: Europace. 2024 Sep 16;26(10):euae239. doi: 10.1093/europace/euae239 (PMC11481322; doi:10.1093/europace/euae239)

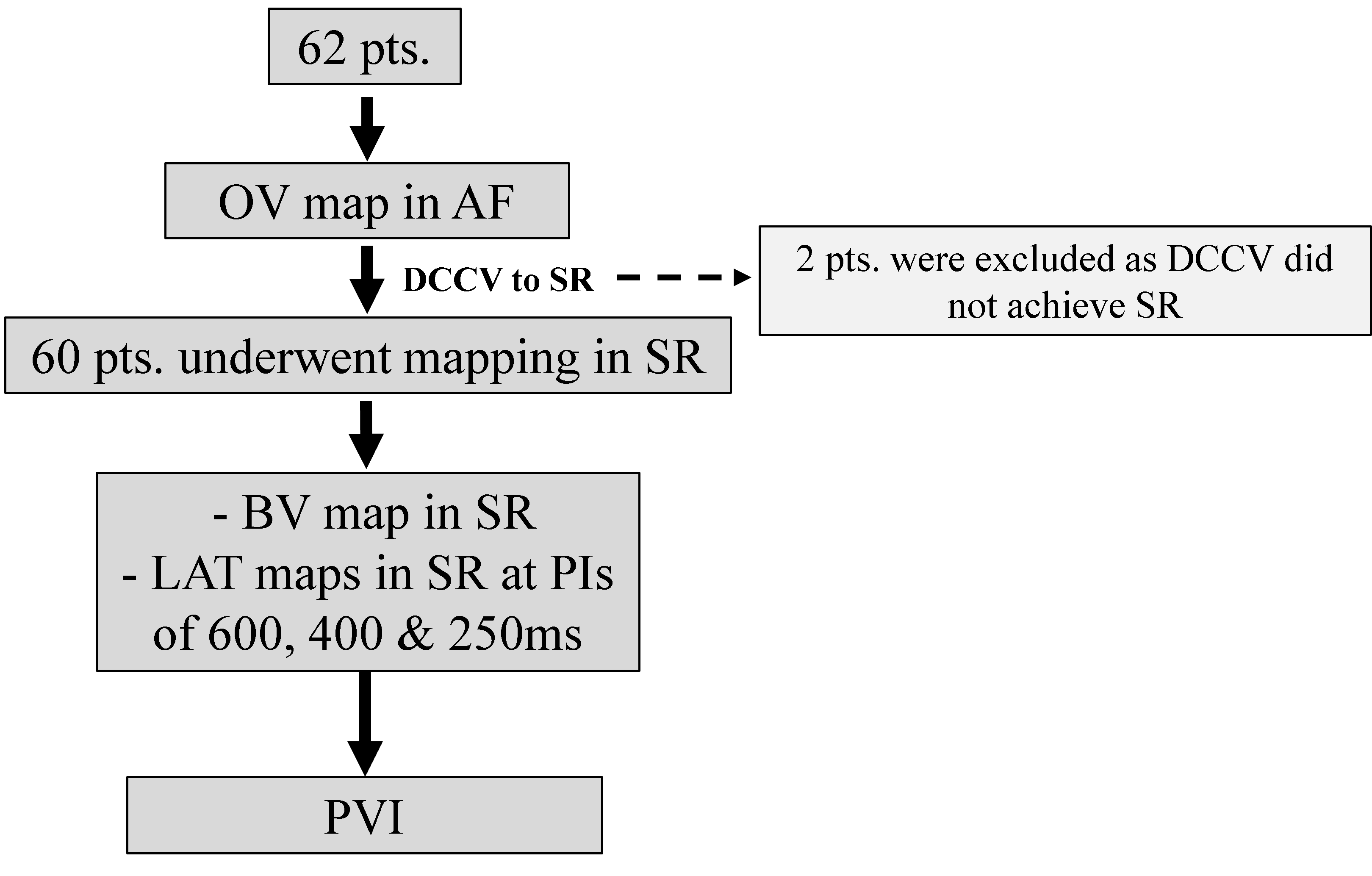

Supplement: euae239_Supplementary_Data [file euae239_supplementary_data.zip › Supplemental Figure 1.tif]

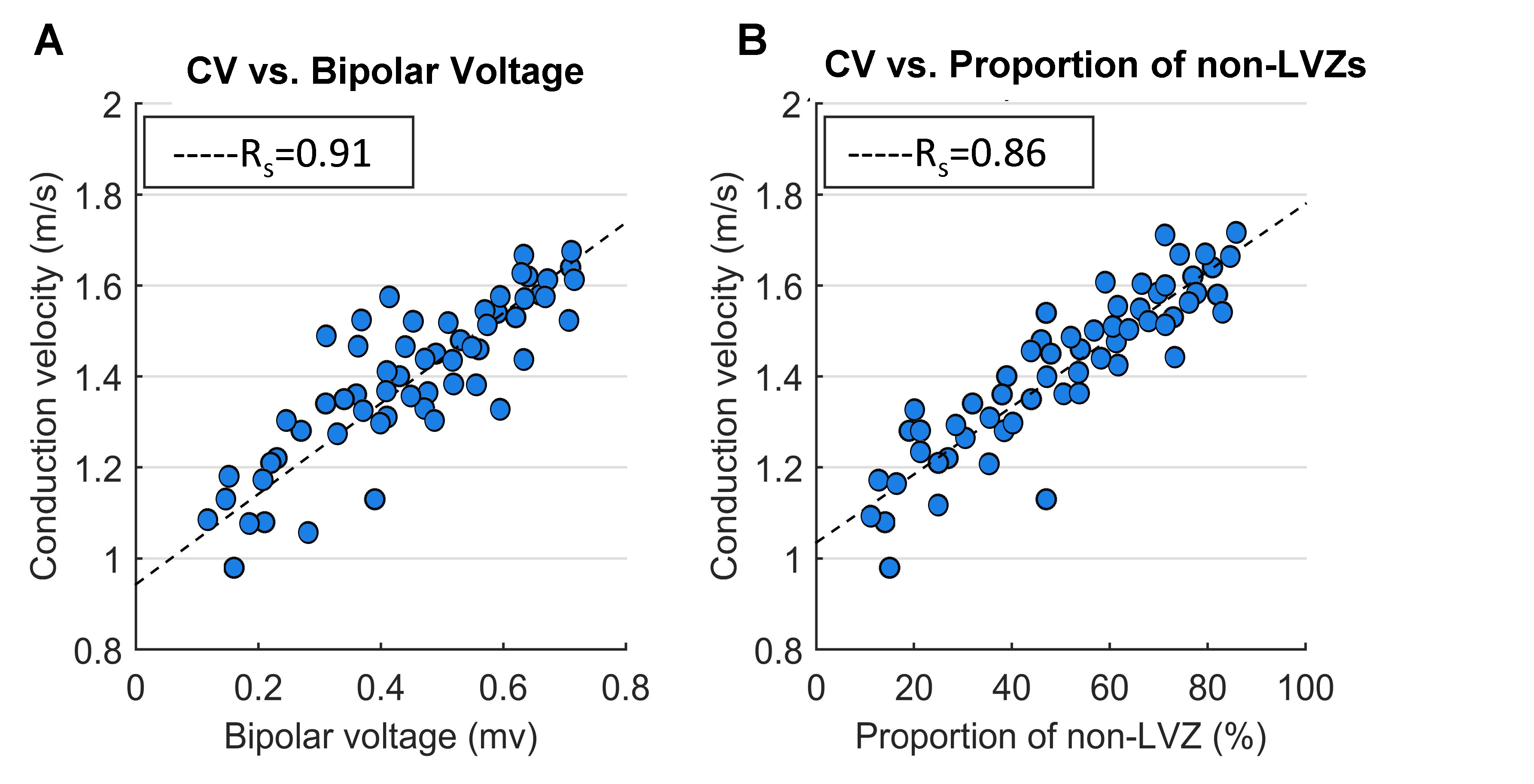

Supplement: euae239_Supplementary_Data [file euae239_supplementary_data.zip › Supplemental Figure 2A-B.tif]
